# Interaction Mechanism of Arc, Keyhole, and Weld Pool in Keyhole Plasma Arc Welding: A Review

**DOI:** 10.3390/ma17061348

**Published:** 2024-03-15

**Authors:** Shinichi Tashiro

**Affiliations:** Joining and Welding Research Institute, Osaka University, Osaka 567-0047, Japan; tashiro.shinichi.jwri@osaka-u.ac.jp; Tel.: +81-6-6879-8666

**Keywords:** plasma arc welding, arc plasma, keyhole, weld pool, interaction, driving force, heat transport, mass transport

## Abstract

The Keyhole Plasma Arc Welding (KPAW) process utilizes arc plasma highly constricted by a water-cooled cupper nozzle to produce great arc pressure for opening a keyhole in the weld pool, achieving full penetration to the thick plate. However, advanced control of welding is known to still be difficult due to the complexity of the process mechanism, in which thermal and dynamic interactions among the arc, keyhole, and weld pool are critically important. In KPAW, two large eddies are generally formed in the weld pool behind the keyhole by plasma shear force as the dominant driving force. These govern the heat transport process in the weld pool and have a strong influence on the weld pool formation process. The weld pool flow velocity is much faster than those of other welding processes such as Tungsten Inert Gas (TIG) welding and Gas Metal Arc (GMA) welding, enhancing the heat transport to lower the weld pool surface temperature. Since the strength and direction of this shear force strongly depend on the keyhole shape, it is possible to control the weld pool formation process by changing the keyhole shape by adjusting the torch design and operating parameters. If the lower eddy is relatively stronger, the heat transport to the bottom side increases and the penetration increases. However, burn-through is more likely to occur, and heat transport to the top side decreases, causing undercut. In order to realize further sophistication of KPAW, a deep theoretical understanding of the process mechanism is essential. In this article, the recent progress in studies regarding the interaction mechanism of arc, keyhole, and weld pool in KPAW is reviewed.

## 1. Introduction

**Figure 1:**
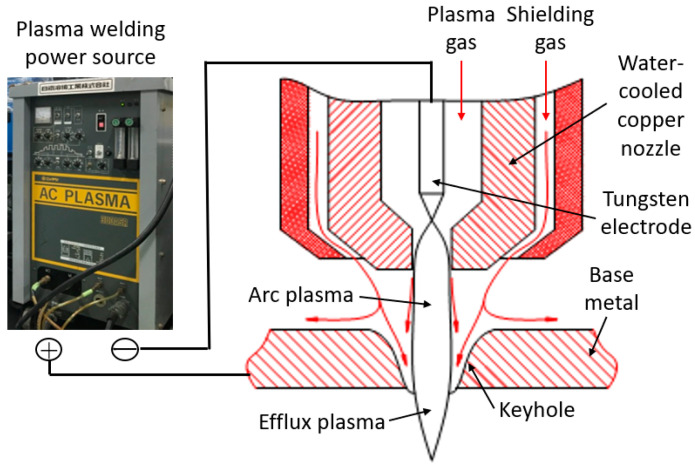
Schematic of plasma arc welding [4].

Plasma Arc Welding (PAW) is a type of arc welding process utilizing a non-consumable electrode [1]. PAW uses a tungsten electrode like Tungsten Inert Gas Welding (TIGW) to generate an arc discharge between the electrode and the base metal. This arc is cooled by a water-cooled copper nozzle attached to the downstream side of the tungsten electrode and is constricted by the thermal pinch effect [2], so that an arc with a high current density can be obtained. Accordingly, the plasma jet is strongly accelerated by the Lorentz force and the plasma temperature is highly increased by Joule heating. The high current density causes strong heat transfer from the arc to the base metal due to electron condensation together with thermal conduction from the high-temperature plasma. Consequently, it is possible to obtain a much higher arc pressure and heat flux to the base metal than TIGW, easily forming deep and narrow penetration. Figure 1 shows a schematic diagram of PAW.

There are two types of welding modes in PAW, melt-in or conduction mode [3] and keyhole mode [1], depending on the operating parameters. In the former mode, the current value and the plasma gas flow rate are suppressed to generate an arc having a relatively low arc pressure and heat flux, so that welding without forming a keyhole is performed. The latter is called Keyhole PAW (KPAW). The base metal is partly melted by the high heat flux from the arc, and the weld pool surface is gradually pushed down by the arc pressure to form a blind keyhole in the base metal and then full penetration can be achieved to form an open keyhole. This mode is mainly used for square butt welding using the I-groove. In this article, the latter mode is reviewed.

When welding steel plates, KPAW enables to weld up to a plate thickness of about 6–10 mm in a single welding pass [5]. In the case of Gas Metal Arc Welding (GMAW) of thick plates, a V- or Y-groove is generally applied to the base metal, and several welding passes are required, because of the insufficient penetration ability of GMAW [6]. On the other hand, KPAW generally uses an I-groove, which does not require operation beveling (groove processing), and the welding can be completed in a single welding pass. This enables high-efficiency welding with reduced man-hours and decreases heat input to the base metal, suppressing thermal distortion. Other advantages are that the capital investment is much cheaper than for laser welding and electron beam welding, the tolerance to joint gap of the base metal is large, and also the electrode consumption is small. Due to the many excellent advantages described above, KPAW has a wide range of applications such as structural steel [7], aerospace [8], and automobiles [9].

Much research has been carried out so far with the aim of improving the quality and efficiency of KPAW [10,11,12]. The weld pool formation process of KPAW is strongly influenced by keyhole behavior. In order to guarantee full penetration, it is generally necessary to maintain an open keyhole state with an appropriate keyhole diameter. The keyhole diameter largely depends on the current and the plasma gas flow rate. If these are insufficient, a blind keyhole is formed, making it difficult to obtain full penetration. On the other hand, if both are excessive, the keyhole diameter becomes large. However, the volume of the weld pool increases accordingly, and the width of the weld pool on the bottom surface increases. As a result, the surface tension cannot support the weight of the molten metal, thus leading to a burn-through. Therefore, in order to solve this problem, it is indispensable to develop sensing and control methods for the keyhole during welding.

Various types of direct or indirect sensing methods for the keyhole have been proposed [10]. For example, the voltage of efflux plasma was measured with a probe installed under the base metal [13]. The voltage signal was found to appear only during the presence of an open keyhole and was enabled to be used for estimating the keyhole diameter. The voltage of plasma cloud above the top surface of the base metal also can be used for monitoring the keyhole state [14]. The plasma cloud is formed by the reflection of plasma jet in the keyhole; so, in the blind keyhole case, the plasma cloud becomes denser. The above methods are classified as indirect sensing. Those are convenient methods, but it is difficult to obtain accurate information on the size, shape, and position of keyhole. Direct sensing using a vision sensor can obtain more accurate information. An ultra-high shutter speed vision system was used to simultaneously image the keyhole and the weld pool from the backside of the work-piece [15]. The camera system includes a strobe-illumination unit (pulse laser), camera head, and system controller. By using this system, both the keyhole and the weld pool are imaged clearly and simultaneously. Wu’s team developed a cost-effective vision system for observing the keyhole from the backside of the work-piece, and clear images of the keyhole are obtained [16]. This vision system can obtain the correlation between the detailed keyhole behavior and welding parameters, so it might provide clues to stabilize the keyhole. A pulse current waveform can be used together with the voltage signal detection system of efflux plasma for controlling the keyhole behavior [17,18]. The pulse parameters are adjusted in real-time referring to the voltage signal, maintaining the “one keyhole one pulse” mode to achieve sound bead formation regardless of changes in the plate thickness during welding [18].

In order to improve the quality and efficiency, variant novel processes of KPAW have been developed [11]. Variable Polarity Plasma Arc Welding (VPPAW) is used to weld aluminum, because it enables to clean an oxide layer on the aluminum surface utilizing cathode spots during the Electrode Positive (EP) period [19]. A novel soft VPPAW has been proposed for decreasing arc pressure acting on the keyhole by using a special nozzle with three holes, suppressing welding defects such as undercut and porosity [20]. Gas focusing PAW was designed for improving penetrability [21]. The focusing gas is used to focus arc plasma which is flowing out of the nozzle, increasing the penetration depth. An ultrasonic vibration-assisted KPAW process was developed [22]. The tungsten electrode connected with a specially designed ultrasonic transducer directly transmitted ultrasonic vibration into the plasma arc. During welding, an open keyhole can be produced with a lower welding current and higher welding speed compared to the conventional KPAW. Plasma–GMA (or Metal Inert Gas (MIG) using inert shielding gas for GMA) hybrid welding is especially suitable for welding of thick plates [23,24]. PAW in the leading position produces a keyhole and then GMAW in the trailing position fills the keyhole with metal droplets transferred from the wire, achieving high-efficiency welding with low heat input. However, the process mechanism of plasma–GMA hybrid welding is known to be very complex especially due to an interaction between two arcs, therefore is difficult to control precisely. Yu et al. tried to control the arc coupling phenomenon by applying an external magnetic field for stabilizing metal transfer [25]. The result showed that the metal transfer mode was significantly affected by the plasma current via the arc coupling. The arc coupling duration enables it to be controlled by the external magnetic field. The influence of the external magnetic field on the welded bead was also investigated [26]. It was found that the mechanical properties of the welded bead were improved by the effect of the external magnetic field. The transport of molten metal provided by GMAW in the weld pool was then studied to discuss the element mixing process [27]. Recently, metal transfer and the weld pool flow in VPPA-MIG hybrid welding of aluminum alloy plates were observed to understand the process mechanism [28].

As described above, various approaches are attempted to improve the KPAW process. However, a deep theoretical understanding of the process mechanism of KPAW is essential to achieve further sophistication. In KPAW, the physical phenomena in the arc, keyhole, and weld pool are strongly coupled via thermal and dynamic interactions. This article reviews the recent progress in studies on the process mechanism of KPAW. Section 1 is the introduction. Section 2 is concerned with plasma arc characteristics. In addition, the effect of metal vapor evaporated form the weld pool is also presented. In Section 3, the keyhole formation process is discussed. Section 4 presents the weld pool formation process. Section 5 provides the summary and future work.

## 2. Plasma Arc Characteristics

First, the plasma arc characteristics of PAW are described. As mentioned in Section 1, PAW generates a plasma arc via electric discharge between the tungsten electrode and the base metal. The current density is increased by contracting the plasma arc with a water-cooled copper nozzle mounted directly under the tungsten electrode. This increase in current density enhances the Lorentz force to accelerate the plasma arc jet from the tungsten electrode to the base metal. At the same time, strong Joule heating occurs and the plasma arc becomes extremely high temperature, especially near the nozzle. The high heat flux brought by the constricted plasma arc starts to melt the base metal, and the molten metal is pushed down by the arc pressure to form a keyhole. As described later, it is considered that the keyhole shape is determined mainly depending on the arc pressure field. When the open keyhole is formed, the plasma arc jet flowing from the electrode side branches in the keyhole. A part of this passes through the keyhole exit and flows down to the bottom side to become efflux plasma, the rest flows outward along the keyhole wall on the top side [29]. Both impose a strong shear force on the surface of the weld pool and are considered to be the main driving force of the weld pool convection in KPAW.

Therefore, in order to control the formation process of the keyhole and the weld pool, it is essential to understand the influence mechanism of the torch design such as the electrode shape, the water-cooled copper nozzle shape, and the electrode setback, as well as the operating parameters such as the current and the composition and flow rate of the plasma gas and shielding gas, on the plasma arc characteristics including the temperature field and velocity field of the plasma arc, as well as the arc pressure, shear force, current density, and heat flux applied to the base metal.

### 2.1. Plasma Temperature

The temperature field of the plasma arc governs the current path in the arc, so it is the most important quantity to affect the heat source properties of PAW. The temperature is mainly acquired by spectroscopic measurement. The measured temperature field is very useful information for validating the numerical simulation results. When performing numerical simulations of plasma arc characteristics, the Local Thermodynamic Equilibrium (LTE) approximation [30] is generally applied for simplicity, but it is known that non-equilibrium phenomena occur, for example, in low-temperature regions where the collision frequency between electrons and heavy species decreases [31]. Since the current path in the plasma arc is strongly affected by a change in electrical conductivity caused by the non-LTE phenomena, it can be an important factor for improving the calculation accuracy.

Boselli et al. spectrally measured the temperature field of PAW in a low-current range of 40 A by the relative line intensity method, and the temperature reached about 14,000 K on the arc axis below the nozzle, which gradually decreased in the radial direction [32]. They also calculated the temperature field using a non-equilibrium model, comparing the result with that of the conventional LTE model and the temperature measurement results. The excitation temperature of argon plasma measured around the outer edge of the plasma arc was consistent with the electron temperature obtained by the non-equilibrium model and was much higher than the LTE temperature, indicating that non-equilibrium phenomena are strongly exhibited in this region. 

Pan et al. proposed a fully coupled tungsten-plasma-anode model for the first time, to study the heat transport and fluid flow during keyhole formation in KPAW [33]. For the calculation, the current was set to 120 A, and the plasma arc was assumed to satisfy the LTE approximation. The radial distribution of electron temperature at the mid-height of the arc gap was measured by the Boltzmann diagram method. As a result, it reached 25,000 K on the arc axis and dropped to almost room temperature at a radius of 3 mm. The measured electron temperature distribution is in good agreement with the calculation result under LTE approximation, indicating that the calculation assuming the LTE approximation is appropriate for a relatively high current range. 

**Figure 2:**
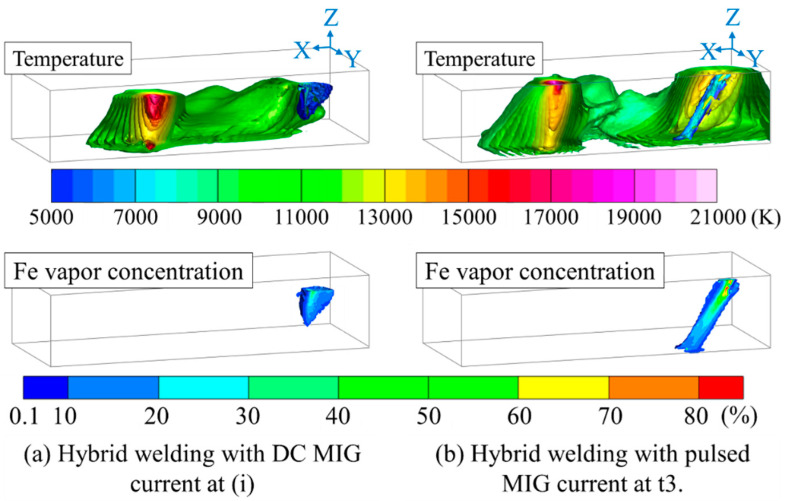
Temperature field and metal vapor concentration field in plasma–MIG hybrid welding obtained by three-dimensional spectroscopic measurement [35].

Ishida et al. obtained the temperature field and metal vapor concentration field in plasma–MIG hybrid welding by three-dimensional spectroscopic measurement [34] and elucidated the coupling mechanism of the plasma arc and MIG arc [35]. When MIGW used pulsed current, the arc coupling occurred only during the upslope of the pulse. It is shown in Figure 2 that the plasma arc temperature at 220 A was about 19,000 K just below the nozzle, but this increased when arc coupling occurred to connect the plasma arc current and MIGW current partly.

### 2.2. Metal Vapor

As the temperature of the weld pool rises, metal vapor evaporates from the surface. When the metal vapor is mixed with the plasma, it has a great effect on its thermodynamic and transport properties and radiation loss [36]. Especially in a low-temperature range of 10,000 K or less, the electrical conductivity of plasma increases by several orders of magnitude even if metal vapor is mixed at only about 1%. In addition, the radiation loss increases remarkably as the mixing rate increases. 

For example, in the case of TIGW of stainless steel SUS304 at 150 A, the iron vapor concentration near the center of the weld pool is about 7% when helium gas is used and about 0.2% when argon gas is used [37]. Particularly, in the former case, it was shown that the current density of the plasma was greatly reduced, decreasing the penetration depth. 

Li et al. investigated the effect of metal vapor in conduction PAW with argon gas at 40 A [3]. Due to the constriction effect of the arc, the iron vapor concentration near the center of the weld pool reached about 9%. As a result, it was found that the thermal conduction from the arc to the base metal was reduced to about half due to the mixing of metal vapor. 

On the other hand, Jian et al. conducted a simulation of the keyhole formation process immediately after the arc ignition when argon KPAW was performed at 150 A considering the influence of metal vapor [38]. Inside the keyhole, the weld pool temperature was low, indicating that there was almost no metal vapor. The temperature of the weld pool increased near the outer edge of the keyhole, and the metal vapor concentration reached 0.2% maximum at that position. 

Consequently, the plasma arc inside the keyhole is hardly affected by the metal vapor, and it is considered that this has little effect on the formation of the keyhole.

### 2.3. Arc Pressure

In variable polarity arc welding, the energy balance on the surface of the tungsten electrode changes greatly depending on the polarity. In the Electrode Negative (EN) phase, the surface tends to be cooled by thermionic electron emission, and in the Electrode Positive (EP) phase, it tends to be heated by electron condensation [39]. Since the electrode surface temperature governs thermionic electron emission characteristics, the current attachment is thought to be greatly affected by the polarity. 

Xu et al. proposed a VPPAW model incorporating a current attachment model considering the above energy balance and clarified the effect of energy balance of the electrode on plasma arc characteristics and also the arc pressure on the base metal [40]. Due to energy balance on the electrode surface, the electrode temperature was decreased over time by thermionic electron emission at the early stage of the EN phase. As a result, the current attachment was constricted to the tip of the electrode. It enhanced the Lorentz force acting on the arc column in the EN phase, making the arc pressure higher. 

### 2.4. Comparison with TIGW

The arc pressure, shear force, current density, and heat flux on the surface of the base metal are considered to be particularly important as inputs from the plasma arc to the base metal, affecting the formation processes of the keyhole and the weld pool. Here, these values are compared with results under similar conditions of TIGW, which is a welding method using a tungsten electrode as in PAW. 

From the results of numerical simulations of argon TIGW at 150 A, the arc pressure was found to be 400 Pa at the arc axis [41] and the shear force was increased up to 40 Pa at a position slightly apart from the axis [42]. In the same condition, the current density and heat flux were measured on the axis to be a maximum of 5 × 10^6^ A/m^2^ and 5 × 10^7^ W/m^2^, respectively [43]. 

On the other hand, in PAW, the arc pressure at 150 A was measured by Xu et al. [44] and Li et al. [21]. It was reported that the maximum values were 3.3 kPa and 3.5 kPa, respectively. The shear force at 135 A was calculated to be about 500 Pa by Wu et al. [45]. The current density and heat flux at 120 A were calculated by Pan et al., as 8 × 10^6^ A/m^2^ and 4 × 10^7^ W/m^2^ [33]. Calculations at 170 A by Jian et al. showed 8 × 10^6^ A/m^2^ and 1.2 × 10^8^ W/m^2^ [46]. 

In summary, it was shown that the arc pressure and shear force of PAW reached about 10-times those of TIGW, and the current density and heat flux were higher than those of TIGW. As mentioned above, these also vary greatly depending on the torch structure and operating parameters.

### 2.5. Effect of Torch Design and Operating Parameters

Due to the difficulty to measure the temperature field and velocity field of the plasma arc, there are few reports of experimental measurement results as described above, so numerical simulation is mainly utilized for the investigation. 

Schnick et al. predicted the plasma arc characteristics in PAW through numerical investigation [47]. Here, the plasma arc is assumed to satisfy the LTE approximation. The base metal is a water-cooled copper anode with a flat surface. The effects of design parameters, gas composition, gas flow rate, and current on the temperature and velocity fields of the plasma arc were clarified. Furthermore, the effects on the arc pressure and heat flux on the base metal have also been elucidated.

Since the arc is constricted by the water-cooled copper nozzle, the Lorentz force works strongly, and the maximum flow velocity of the argon plasma arc with a current of 100 A reaches about 1500 m/s. This velocity is about 7.5-times the calculation result of TIGW at a current of 150 A [48]. The arc pressure and shear force acting on the base metal increase as the flow velocity of the plasma arc increases. In addition, the temperature of the plasma arc reached a maximum of 25,000 K directly under the tungsten electrode due to an increase in Joule heating. This is about 1.5-times the measurement result of TIGW at a current of 100 A [49]. 

Investigations also show that an increase in current raises the plasma temperature and plasma flow velocity, while the plasma gas flow rate hardly affects the plasma temperature but increases the plasma flow velocity. However, both increase the heat flux and the arc pressure at the base metal. The nozzle diameter and cathode position are adjustable to affect the plasma arc. The larger torch distance expands the plasma arc column gradually due to the effect of the high viscosity of plasma, thus decreasing the maximum arc pressure. On the other hand, the internal angle of the plasma nozzle is less effective. The effect of gas composition was also discussed. The addition of helium to argon increases the viscosity of plasma arc, giving a dominant effect on decreasing the arc pressure. The addition of hydrogen raises the thermal conductivity, increasing the heat flux. According to the demixing effect, the concentration of helium and hydrogen increases around the arc axis in the vicinity of the base metal surface.

## 3. Keyhole Formation Process

Next, the keyhole formation process is described. Since the energy and force from the plasma arc are brought to the base metal through the keyhole wall, these are strongly affected by the shape and stability of the keyhole, so a deep understanding of the keyhole formation process is required. 

After the arc ignition, the surface of the base metal begins to melt due to the high heat flux from the plasma arc. The surface of the weld pool is pushed down by the arc pressure, and the molten metal is transported to the outside by the weld pool convection driven by the shear stress due to the plasma flow and the Marangoni force caused by the temperature gradient on the weld pool surface, thus forming a blind keyhole. It is considered that the keyhole shape is mainly determined by the force balance between the arc pressure acting to expand the keyhole and the surface tension and gravity acting to close it. As the keyhole depth increases, the heat flux and arc pressure applied to the deepest part gradually decrease, but if those are large enough, the keyhole penetrates to the bottom to form an open keyhole. 

In keyhole welding using a laser or electron beam, the weld pool temperature inside the keyhole rises maximally up to around the boiling point. Therefore, the evaporation of metal vapor is known to be significantly intensive, so the recoil pressure accompanying this evaporation is the main driving force for the keyhole formation [50]. On the other hand, in case of KPAW, since the surface temperature of weld pool is just above the melting point, the evaporation of metal vapor is very weak [38], so it is considered that the recoil pressure due to evaporation is negligibly small.

### 3.1. Keyhole Formation Models

In the study of the formation process of the keyhole and the weld pool around it, in many cases, the heat transport process is investigated mainly by numerical simulation supported by experimental observation. Since it is difficult to directly observe the dynamic keyhole phenomenon during welding, analysis by numerical simulation is effective. Therefore, various types of simulation models have been proposed so far. 

Investigations using simple arc heat source models [51,52] have been carried out since the early stages of the research. Models were improved to follow dynamic changes in the keyhole shape. In simulation using the arc heat source model, source terms described with special functions are generally given to the energy conservation equation and momentum conservation equation for considering the effects of heat flux, arc pressure, shear force, etc., from the arc. In conjunction with this model, the Volume of Fluid (VOF) method is often used to track the weld pool surface [53,54,55,56]. Some constants and coefficients of functions used in the arc heat source model are empirically determined based on the experimental results. This model has a low calculation cost and is highly convenient from an engineering point of view. It is mainly suitable for predicting the dependence of experimental parameters on the weld pool shape and penetration shape. 

On the other hand, in recent years, in order to study the thermal and dynamic interactions of the arc, keyhole, and weld pool in more detail, a coupled electrode-arc-keyhole-weld pool model applying the VOF method has been developed [33,46,57,58]. This model does not require various assumptions used in the arc heat source model and can comprehensively clarify the effects of the torch design and operating parameters on the formation of keyholes and weld pools through changes in arc characteristics. Therefore, compared with the arc heat source model, it becomes possible to examine the keyhole formation process in more detail. However, it is computationally very expensive and difficult to apply it to a large-scale parametric study. 

Furthermore, a simple coupled model [29,59] using a pre-fixed keyhole shape without tracking the weld pool surface by the VOF method is proposed to reduce calculation costs.

A one-way coupling model [45] has also been proposed, in which the arc model and the weld pool model are not coupled in a two-way, but the boundary conditions of the weld pool are determined based on arc characteristics obtained by the arc model.

### 3.2. Driving Force for Keyhole Formtion

Wu et al. used the aforementioned model to conduct a detailed study of the keyhole formation process [60]. In welding, arc pressure, shear force, Marangoni force, etc., which are the driving forces for keyhole formation, work simultaneously. But, by giving these forces individually as virtual forces, the dominant driving force for keyhole formation can be examined. Consequently, it is found that the weld pool deformations driven by the arc pressure and shear stress are suggested to be the two responsible mechanisms for the formation of keyhole. The first one is especially dominant.

The process between full penetration of the weld pool and the formation of an open keyhole is defined as the blasting penetration stage, where the molten bridge located at the bottom of the blind keyhole rapidly breaks [54,61]. As a result of numerical simulation [45], the pressure balance and also the mass conservation mechanisms are considered to strongly affect the occurrence of blasting penetration. The decrease in the melting point and surface tension as well as the deformation of the bottom surface are thought to facilitate the blasting penetration. On the other hand, the increase in thermal conductivity restraints the blasting penetration.

### 3.3. Energy Balance

The energy balance between the plasma arc and the weld pool through the keyhole wall has also been studied by numerical simulation. 

In KPAW, after the open keyhole formation by plasma arc, part of the plasma becomes an efflux plasma that blows out below the base metal through the keyhole exit. Li et al. estimated that about 10% of the plasma arc outflows from the keyhole exit [29], even though this value is predicted to change depending on the torch design and operating parameter.

**Figure 3:**
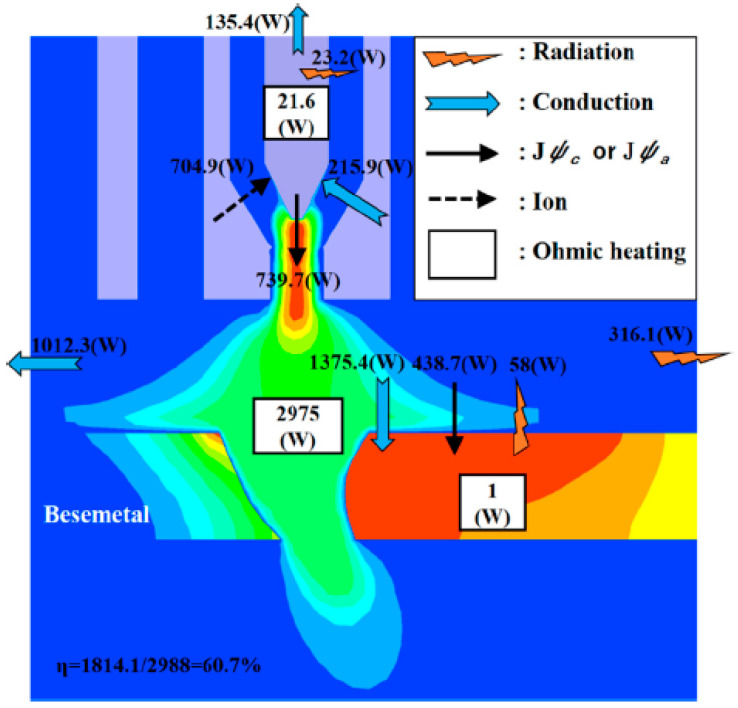
The energy balance in the KPAW process [62].

Wu et al. studied the energy balance in KPAW [62,63]. The energy transport among the electrode, plasma arc, and base metal was numerically evaluated using the coupled electrode-arc-keyhole-weld pool model applying the pre-fixed keyhole. Figure 3 shows the energy balance in the KPAW process of steel plate [62]. Thermal efficiencies of the PAW process with and without keyhole were also compared. In the case with a keyhole, both the energy consumption by arc and the energy input increase. On the other hand, the total energy transport from the arc to the weld pool slightly decreases, thus the thermal efficiency decreases. The open keyhole formation suppresses the energy transport to the weld pool, and 11.5% of the arc energy was lost by the efflux plasma. The calculated thermal efficiency is only 60.7% [62]. The thermal efficiency of KPAW was found to be relatively lower than those of GTAW and GMAW processes. Tanaka et al. presented the thermal efficiency of 82% in the GTAW process [41]. Joseph et al. experimentally obtained a thermal efficiency of 68–72% in the pulsed GMAW process through liquid nitrogen calorimetry [64].

### 3.4. Relaationship between Keyhole Exit Deviation and Porosity Formation

Wu et al. clarified the effect of keyhole behaviors on the formation of porosity in KPAW [65,66]. From the observed image of the keyhole exit, the dimensional parameters as well as the keyhole exit deviation distance were obtained. Pulsed PAW was carried out for bead-on-plate welding and closed butt joint welding. The result showed that in the case of a large keyhole exit deviation distance, high reinforcement was formed in the front weld surface to induce porosity in the solidified weld, while in the case of a small distance, a sound weld was achieved. The keyhole exit deviation distance is suggested to be a critical keyhole factor to determine the weld quality. In a keyhole with a large deviation distance, the gas flow field was disturbed and much liquid metal was pushed toward the front pool surface. Consequently, the weld pool was deformed largely with an inside gas cavity. The results indicate that the weld quality in KPAW highly depends on the keyhole behavior.

### 3.5. Effect of Torch Design and Operating Parameters

The keyhole shape after open keyhole formation has a strong effect on the energy and force balance between the plasma arc and the weld pool. It is suggested that this keyhole shape strongly depends on the torch design and operating parameters as described above. Nguyen et al. studied the effects of plasma gas flow rate [67], current [68], and oxygen concentration in shielding gas [69] in KPAW of a steel plate. In addition, Xu et al. clarified the effects of electrode setback [70] and plate thickness [71] in VPPAW of aluminum alloys. Here, the results for KPAW of a steel plate are shown as an example.

**Figure 4:**
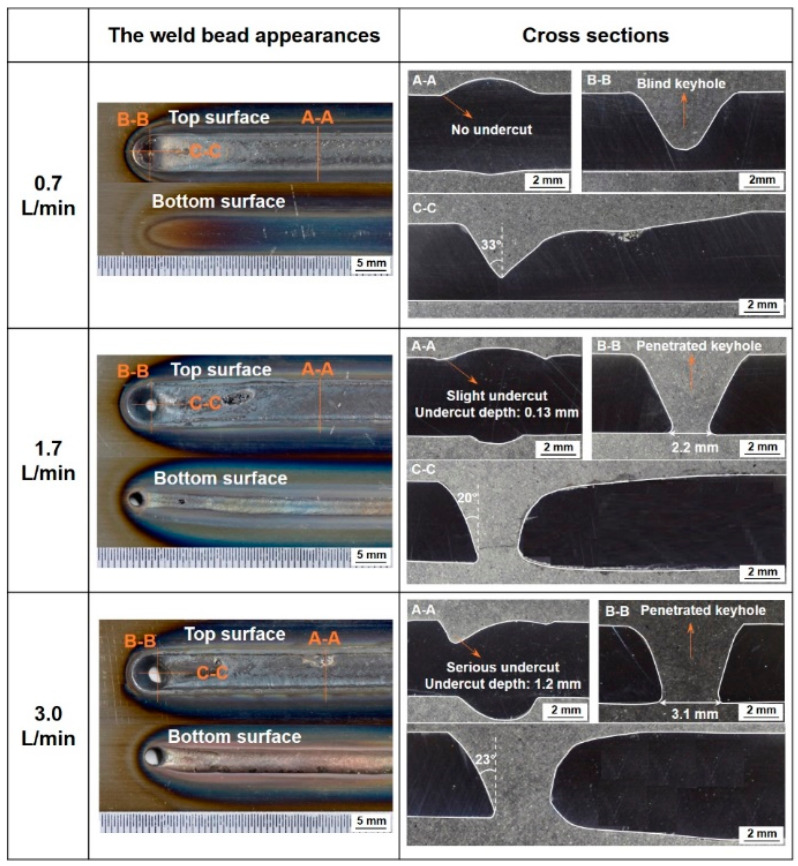
Weld bead appearances and cross sections of the weld beads for different plasma gas flow rates [67].

Figure 4 shows the effect of plasma gas flow rate on the keyhole shape [67]. It can be seen that at a flow rate of 0.7 L/min, the keyhole does not penetrate and becomes a blind keyhole, and at a flow rate of 1.7 L/min or more, it becomes an open keyhole. Under the condition that an open keyhole is formed, as the flow rate increases, the diameter of the bottom keyhole increases and the keyhole wall behind the keyhole becomes nearly vertical. These changes are considered to be due to the remarkable increase in arc pressure due to the increase in gas flow rate. It can be also seen that an undercut occurs at the weld toe when the plasma gas flow rate is large.

**Figure 5:**
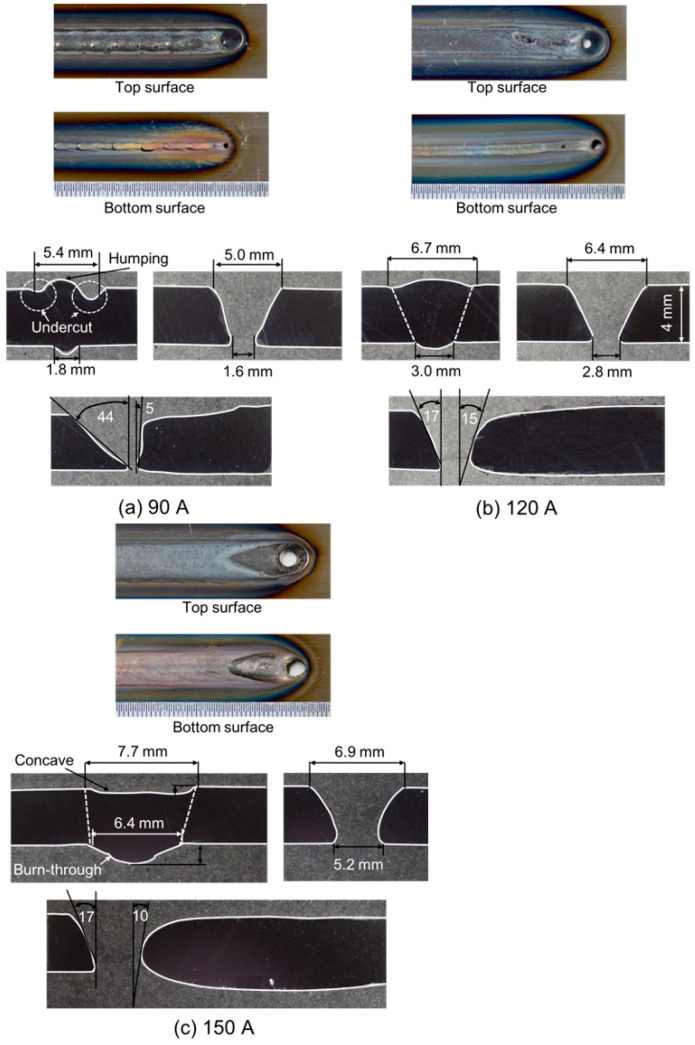
Weld bead appearances and cross sections of the weld beads for different currents [68].

Figure 5 shows the effect of current on the keyhole shape [68]. An open keyhole is formed at all current conditions. At 90 A, the diameter of the bottom keyhole is slightly small with a value of 1.6 mm; but, at 120 A, it expands to 2.8 mm, and at 150 A, it reaches 5.2 mm. At 150 A, the angle of the keyhole wall behind the keyhole is smaller than that at 120 A. In addition, the weld pool width increases with the current. When the current is increased, the amount of heat input is increased, and the electromagnetic force applied to the plasma arc is strengthened, so the arc pressure is also increased. It can be seen that the result at 90 A shows humping on the top surface and that at 150 A shows burn-through on the bottom surface. A sound weld bead is obtained only at 120 A.

**Figure 6:**
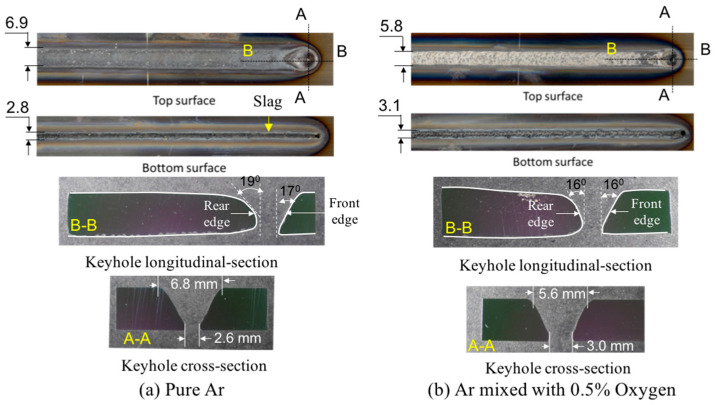
Weld bead appearances and cross sections of the weld beads for different shielding gas compositions [69].

Figure 6 shows the effect of oxygen concentration in the shielding gas on the keyhole shape [69]. Here, a small amount of oxygen is mixed in the shielding gas, assuming shielding failure. The addition of 0.5% oxygen to the argon shielding gas increases the diameter of the bottom keyhole and decreases the angle of the keyhole wall behind the keyhole. This is probably because the surface tension of the weld pool decreased due to the absorption of oxygen into the weld pool. 

As described above, the keyhole shape changes greatly with variations in welding conditions. This has a strong effect on the weld pool formation process as seen in the next section.

## 4. Weld Pool Formation Process

The weld pool formation process is described in this section. During the travel of the torch in the welding direction, the molten metal on the front side of the keyhole is transported through the sides of the keyhole toward the rear region to form the weld pool. The heat transport in the weld pool is considered to be primarily due to weld pool convection, especially in steel welding, driven by the buoyancy force, Lorentz force, Marangoni force, and plasma shear force. Particularly, the shear force and Marangoni force are thought to strongly depend on the keyhole shape, since those work on the surface of the weld pool in the vicinity of the keyhole. The molten metal in the weld pool is supported by the surface tension of the bottom surface of the weld pool, preventing burn-through. As discussed in the previous sections, the arc, keyhole, and weld pool strongly interact together, thus making KPAW a very complex process. Furthermore, the weld pool formation easily becomes unstable, inducing various welding defects, for example, by disturbance or a change in the condition in welding. Accordingly, to achieve high-quality welding, it is necessary to deeply understand the weld pool formation processes.

### 4.1. Weld Pool Observation

Information on the flow field and temperature field is indispensable for understanding the heat transport process in the weld pool, but due to the difficulty in measurement, there are still few reports of these experimental measurement results.

Simultaneous measurement of both the keyhole behavior and its surrounding temperature field of the weld pool is indispensable for understanding the thermo-physical mechanism and also achieving the process control of KPAW. Zhang et al. used an infrared camera and a coupled charge device (CCD) camera to observe the temperature profile of the weld pool in the vicinity of the keyhole and the keyhole behavior from below the base metal at the same time [72]. After image processing and calibration, the shapes and sizes of keyhole and weld pool as well as their relative positions were determined with temperature field.

Chen et al. observed the flow field on the surface of the weld pool employing a high-speed camera equipped with a laser light system and also the temperature field using an infrared thermal camera in VPPAW of aluminum alloys [73]. The formation mechanism of the keyhole weld pool at different welding positions was studied, especially for clarifying the effect of gravity on material flow, temperature field, and keyhole morphology. Based on the result of both calculation and experiment, the effect of gravity on the weld pool was found to be second only to shear force in all factors. Consequently, the heat and mass transport in the low-velocity region of the VPPA keyhole weld pool are thought to be readily influenced by the gravity.

In order to gain a deep understanding of the heat transport process in the weld pool, it is necessary to measure the flow field of the entire weld pool including the inside. For the first time, Nguyen et al. applied stereo synchronous imaging of tungsten tracer particles employing two sets of X-ray transmission systems to measure the 3D flow field inside the weld pool in KPAW [74]. The 2D convection on the surface of the weld pool was also measured by observing the motion of zirconia tracer particles. Based on this comprehensive measurement, the weld pool convection in the region behind the keyhole to the weld pool end was totally visualized. For discussing the heat transport process in the weld pool, the 2D temperature field on the top and bottom surfaces of the weld pool was also obtained by two-color pyrometry. A summary of the results of this comprehensive measurement is provided below.

**Figure 7:**
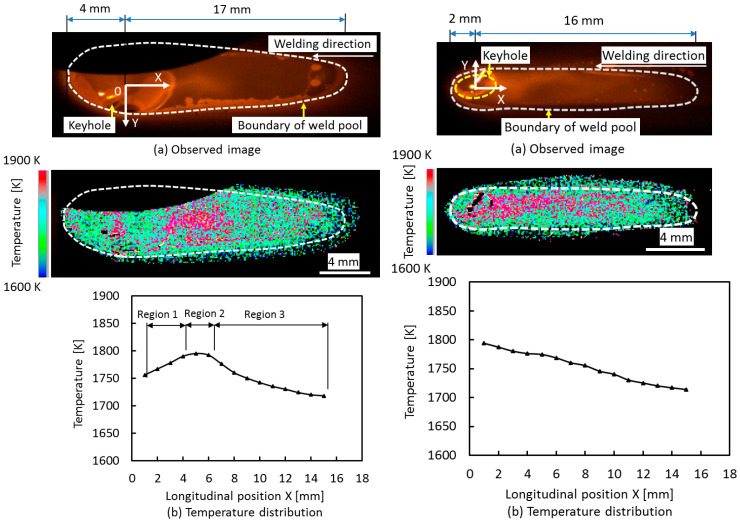
Observed image and temperature field on the surface of the weld pool. (**a**) Observed image; (**b**) temperature field on top (**left** figure) and bottom (**right** figure) surfaces [74].

Figure 7 shows weld pool images and 2D temperature fields on the top and bottom surfaces. On the top weld pool surface, the temperature was measured to be between 1718 K and 1795 K. The weld pool is divided into regions 1–3, according to the difference in temperature. The temperature reached 1795 K in region 2. On the bottom weld pool surface, the temperature was distributed between 1714 K and 1794 K. The temperature monotonically decreased from 1794 K around the keyhole to 1714 K at the weld pool end. 

**Figure 8:**
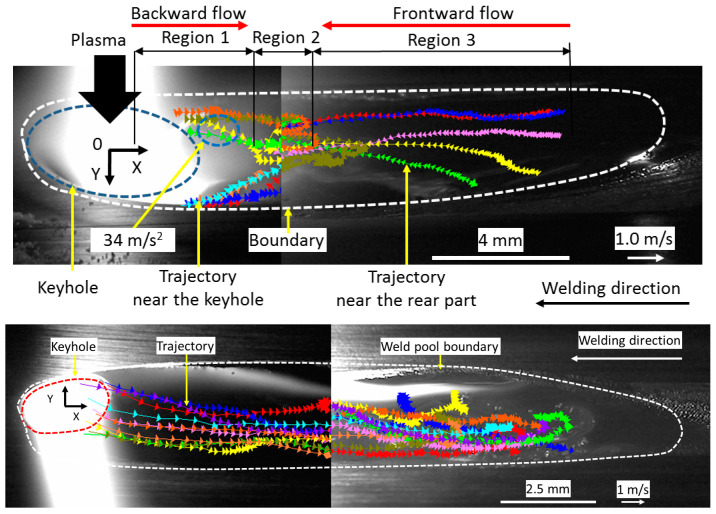
Convective pattern on top (**upper** figure) and bottom (**lower** figure) surfaces of the weld pool [74].

Figure 8 shows 2D convective patterns on the top and bottom weld pool surfaces. On the top weld pool surface, the definition of each region is the same as that in Figure 7. In region 1, molten metal flowed from the keyhole to region 2 at a high temperature. It also flowed inward from the weld pool edge to the center of the weld pool. In region 3, the molten metal flowed in a forward direction from the weld pool end to region 2 at a high temperature. In region 2, the molten metal flows from regions 1 and 3 were merged. Zirconia particles were rotated due to this convective pattern. On the bottom weld pool surface, the molten metal flowed backward to the weld pool end. The maximum velocities of 0.53 m/s and 0.81 m/s were seen at X = 2.0 mm on the top surface and X = 1.0 mm on the bottom surface, respectively.

**Figure 9:**
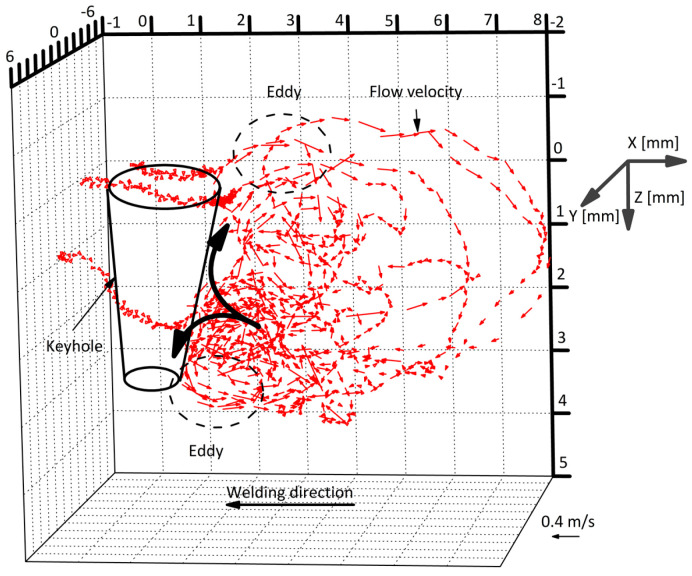
Three-dimensional convective patterns inside the weld pool in KPAW of stainless steel [74].

Figure 9 shows 3D convective patterns in the weld pool. The result showed that two eddy convective patterns in opposite directions were stably formed in the weld pool behind the keyhole. The maximum velocities for the molten metal flows near the top and bottom surfaces reached 0.35 and 0.30 m/s, respectively. The velocity of molten metal flow around the middle height decreased to 0.15 m/s. 

The dominant driving force and heat transport process considered based on the above comprehensive measurement are discussed in Section 4.2.

**Figure 10:**
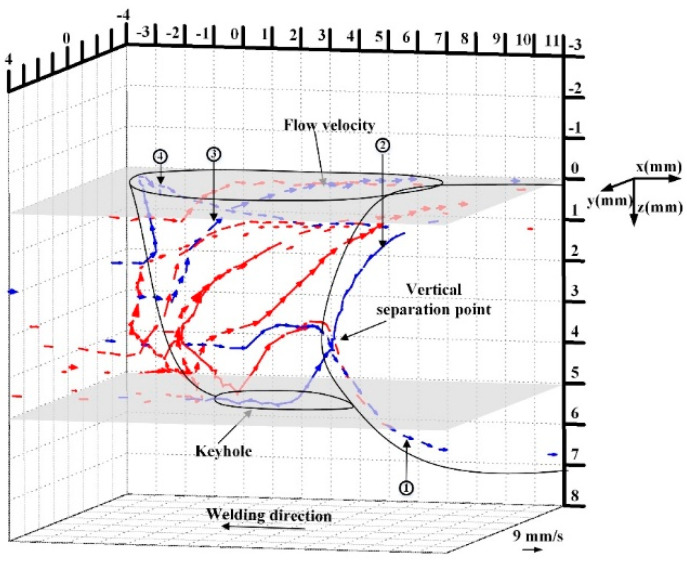
Three-dimensional keyhole detouring flow inside the weld pool in VPPAW of aluminum alloys [75].

Xu et al. clarified the 3D keyhole detouring the flow inside the weld pool in VPPAW of an aluminum alloy employing two sets of X-ray transmission systems [75]. Furthermore, measurement of the surface flow of the weld pool at different regions of the keyhole was also carried out by observing tracers. As a result, it was shown that a thin liquid metal layer was formed on the keyhole boundary because of the high thermal conductivity of the aluminum alloy. Moreover, the liquid metal was mainly formed around the keyhole bottom. Figure 10 shows 3D convective patterns in the weld pool. The liquid metal flowed upward and around the keyhole. The molten metal at the front wall of the keyhole around bottom detoured the keyhole backward. After that, it was blanched into upward and downward flows, forming a vertical separation point. In addition, comparing with the stainless steel case [74], the flow velocity of VPPAW was found to be much smaller.

### 4.2. Dominant Driving Force of Weld Pool Convection and Heat Transport Process in Weld Pool

**Figure 11:**
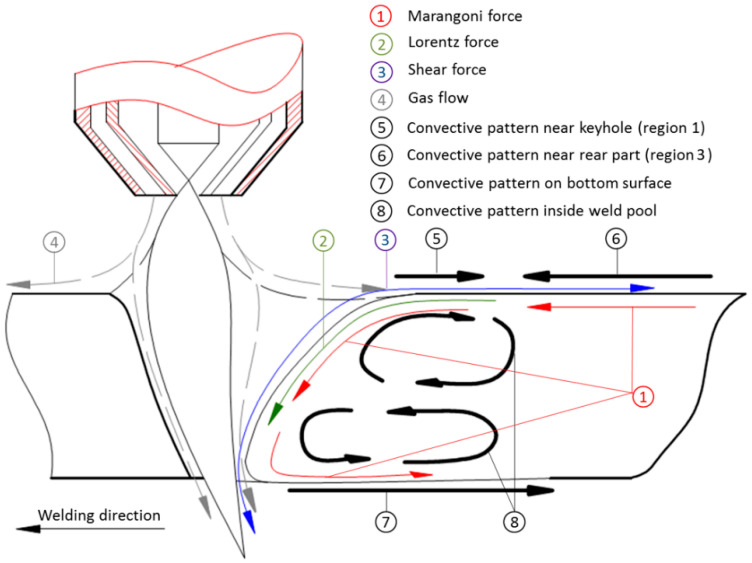
Schematic illustration of the main driving forces and convective patterns of the weld pool [74].

Following the experimental results on KPAW of stainless steel [74] described in Section 4.1, the dominant driving force of the weld pool convection in KPAW and its influence on the heat transport in the weld pool are discussed. As in the TIG welding case [41], the main driving forces of the weld pool convection in KPAW are buoyancy, Lorentz force, Marangoni force, and shear force especially in a quasi-steady state after stable open keyhole formation. Figure 11 presents convections obtained by the measurement and also the estimated driving forces acting on each convection.

The buoyancy is weaker comparing with other forces and acts in an upward direction. Therefore, this force is considered to be negligible for weld pool formation. 

The Lorentz force is generally in an inward and downward direction around the surface of the weld pool, since the current expands conically.

The shear force acting on the surface of the weld pool is composed of two forces. The first one is caused by plasma flowing along the top weld pool surface. The direction of this force is mainly upward and outward, inducing the clockwise convection around the top surface. The second one is caused by plasma flowing out downward through the keyhole exit. The direction of the force is downward, forming the counterclockwise convection around the bottom surface.

The Marangoni force is produced by the gradient of weld pool surface temperature. This force is generally directed from high temperature to low temperature in case of inert shielding gas. On the other hand, if an active gas like oxygen is mixed a little with the shielding gas, as expected in region 3, the force direction is reversed [76]. 

In region 1, the molten metal flowed backward from behind the keyhole to region 2 at a high temperature. The backward shear force was found to surpass the resultant Marangoni and Lorentz forces in the forward direction, therefore considered the dominant force. The maximum velocity appeared in the backside of the keyhole. It decreased as region 2 was approached.

In region 3, the molten metal flowed forward from the weld pool end to region 2 at a high temperature. The forward Marangoni force and backward shear force are considered to mainly act in this region, but the shear force is thought to be lower than the Marangoni force, because the velocity of plasma flow apart from the arc is largely decreased. Therefore, the molten metal flowed in the forward direction. 

In region 2, the molten metal flowing from regions 1 backward and 3 forward merged. 

On the bottom surface, the molten metal flowed backward from the keyhole to the weld pool end. The flow was accelerated by the downward shear force acting on the keyhole wall and backward Marangoni force on the bottom surface of the weld pool. Because of the acceleration by both forces, the flow velocity on the bottom surface was much higher than that on the top surface.

Inside the weld pool, two eddies were seen behind the keyhole. The flow in the upper eddy was in a clockwise direction. This eddy was accelerated by the upward and backward shear forces and the forward Marangoni force. Because the shear force is thought to be significantly larger than the Marangoni force, this eddy is mainly accelerated by the shear force. The flow in the lower eddy was essentially accelerated by the downward shear force and the downward and backward Marangoni forces. Therefore, this eddy flowed in a counterclockwise direction. 

The formation process of the weld pool is also investigated in other arc welding processes through the measurement of velocity field of weld pool convection by tracer particle observation. For instance, the velocity field of the weld pool surface in TIGW was measured by Matsuda et al. [77]. They clarified that the velocity reached around 0.04 m/s around the foot point of the arc axis. Zong et al. measured the velocity field in GMAW and found that the range of velocity was about 0.15–0.20 m/s around the arc and 0.04 m/s around the weld pool end [78].

The weld pool formation process of KPAW is considered to be greatly different from those of TIGW and GMAW. The shear force, Lorentz force, and Marangoni force are known to play a major role as driving forces in TIGW and GMAW cases. In case of KPAW, the magnitudes of the Lorentz force and Marangoni force are predicted to have almost similar levels with those of TIGW and GMAW in the same current condition. On the other hand, the shear force is considered to be much larger compared with those of TIGW and GMAW due to the greater velocity of plasma flow [47], becoming the dominant force. Consequently, the velocity of convective flow surpasses those of TIGW and GMAW. 

**Figure 12:**
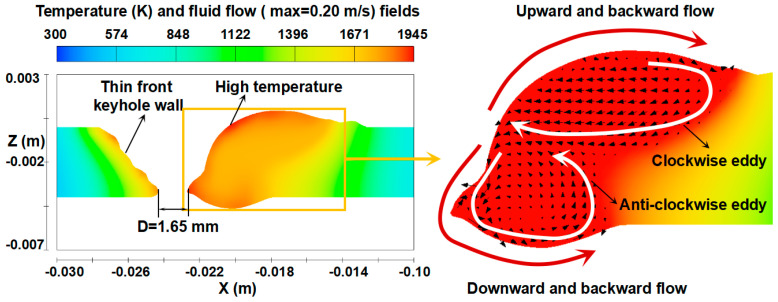
Calculated temperature and fluid flow fields [60].

Wu et al. quantitatively analyzed the weld pool formation process through numerical simulation, presenting that after open keyhole formation, two convective eddies are produced behind the keyhole as in Figure 12 [60]. In the weld pool, the molten metal was transported backward on the top surface by the upper eddy and backward at the bottom surface by the lower eddy. The calculated weld pool convections well agreed with the above observation.

**Figure 13:**
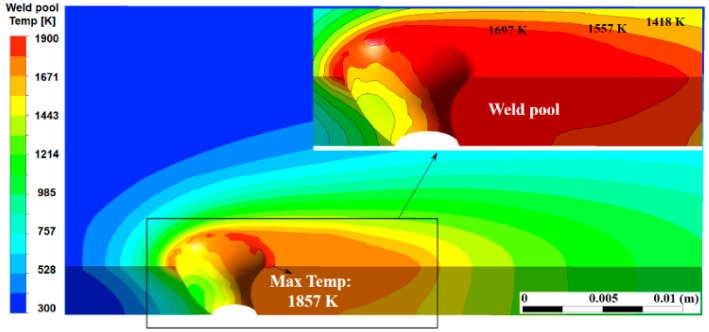
Three-dimensional temperature field in quasi-steady state [62].

Wu et al. carried out a numerical simulation to investigate the heat transport in a weld pool with an open keyhole in a quasi-steady state [62]. Figure 13 shows the 3D temperature field of the weld pool. Although the weld pool temperature is found to increase around the keyhole, the maximum temperature is only 1857 K, which is slightly higher than the melting point of steel. The heat flux from arc to base metal in the KPAW process is large due to the constricted arc, but the weld pool temperature is low. As presented by Nguyen et al. [74], the maximum surface temperature of the weld pool was approximately 1800 K, which was almost the same as that in the moving GTAW [79] and lower than that in the stationary GTAW [80]. The reasons to cause this low temperature can be explained as follows.

Heat transport in the weld pool is carried out mainly by convection and conduction. The Peclet number is useful for discussing the dominant heat transport mechanism. As seen in Figure 9 and Figure 12, two convective eddies were formed behind the keyhole consisting of the upper clockwise eddy and lower counterclockwise eddy. The molten metal was transported backward on the top and bottom surfaces. In the above result, a maximum velocity of 0.2884 m/s was seen in the weld pool. The calculated Peclet number from the velocity was approximately 319. Wang et al. calculated a Peclet number of the weld pool of about 77 in double electrodes TIGW [81]. Generally, when the Peclet number exceeds 10, the effect of heat transport due to convection is considered to be dominant. The result indicates that the heat transport by fluid flow is the dominant mechanism in the KPAW weld pool. As a result, the energy is not accumulated in the weld pool around the keyhole, leading to a lower weld pool temperature.

### 4.3. Effect of Torch Design and Operating Parameters

In the above paper [74], only one experimental condition was performed. However, it is considered that the driving force balance to govern the weld pool formation process significantly depends on the torch design and operating parameters. Because the shear force is strongly affected by the plasma flow field in the vicinity of the keyhole, the force balance is thought to greatly change depending on the keyhole shape. As shown in Figure 9, two large eddies are produced behind the keyhole in this experimental condition, because the plasma flow from the torch is separated to a downward flow outflowing to the bottom side through the keyhole exit and a horizontal and upward flow along the weld pool surface to form two opposite shear forces. For example, in the case of higher plasma gas flow rate or current, the arc pressure acting on the surface of the weld pool is enhanced, leading to a wider keyhole diameter and a steeper keyhole wall. In this case, the downward flow through the keyhole exit might be larger than the horizontal and upward flow. The formation of eddies is expected to be affected by an increase in shear force, making the lower eddy relatively larger. It also causes a variation in the heat transport in the weld pool. Nguyen et al. studied the effect of plasma gas flow rate [67], welding current [68], and also the shielding gas composition [69], and Xu et al. clarified the effects of electrode setback [70] and plate thickness [71] in VPPAW of aluminum alloys. Those studies used the same comprehensive experimental approach.

**Figure 14:**
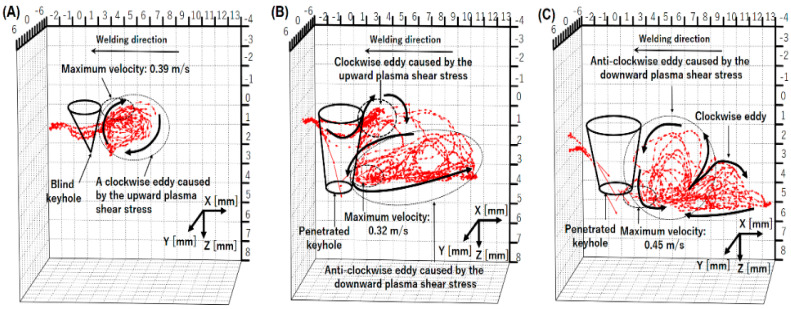
Three-dimensional convective patterns inside the weld pool for different plasma gas flow rates: (**A**) 0.7 L/min; (**B**) 1.7 L/min; (**C**) 3.0 L/min [67].

Figure 14 shows 3D convective patterns inside the weld pool for different plasma gas flow rates [67]. The weld bead appearances and cross sections of the weld beads were already presented in Figure 4. As the plasma gas rate is increased, a penetrated keyhole is formed. The counterclockwise eddy inside the weld pool becomes large and then dominant. The backward flow on the top surface is weakened, while the inward flow is induced by the teardrop-shaped top weld pool profile. According to the dominant counterclockwise eddy inside the weld pool, the molten metal temperature at the lateral sides of the top weld pool is low.

As described above, convection is the dominant mechanism for the heat transport inside the weld pool in KPAW. The strong counterclockwise eddy formed behind the keyhole and the weak backward flow on the top surface of the weld pool produce the uneven energy distribution between the top and bottom surfaces. The strong inward flow causes the uneven energy distribution between the lateral sides and centerline of the top weld pool. All these reasons contribute to the undercut formation at the top surface.

**Figure 15:**
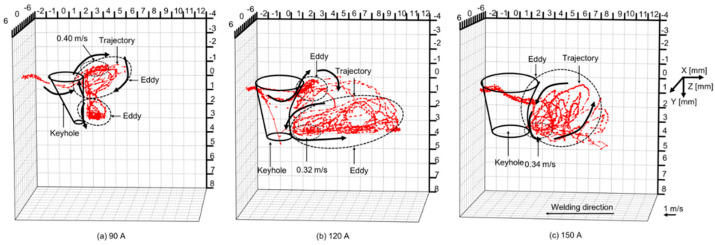
Three-dimensional convective patterns inside the weld pool for different currents [68].

Figure 15 shows 3D convective patterns inside the weld pool for different currents [68]. The weld bead appearances and cross sections of the weld beads have already been presented in Figure 5. In case of a low current of 90 A, an open keyhole was formed with a smaller diameter on the bottom surface than that on the top surface. This is due to the insufficient penetration ability and is considered to produce a strong upward shear force. Therefore, the upper eddy grew up more compared with the lower one. On the other hand, in case of a high current of 150 A, an open keyhole was formed with a large diameter on the bottom surface. The lower eddy was enhanced due to a strong downward shear force. This relative magnitude of the two eddies is thought to significantly affect the heat transport in the weld pool and also the welding defect occurrence. The stronger upper eddy due to the insufficient heat input might cause an undercut and high reinforcement on the top surface. On the contrary, the stronger lower eddy leads to deeper penetration by the large heat transport in a downward direction. However, if the downward heat transport is excessive, it leads to welding defects such as concaves on the top surface and burn-throughs on the bottom surface. Consequently, it is considered that when the arc has sufficient penetration ability to the base metal, the relative magnitude of the two eddies should be suitably controlled for achieving the larger counterclockwise eddy and smaller clockwise eddy, which enables achievement of a stable and high-quality welding preventing welding defect occurrence, as in the optimal current of 120 A in this experiment.

**Figure 16:**
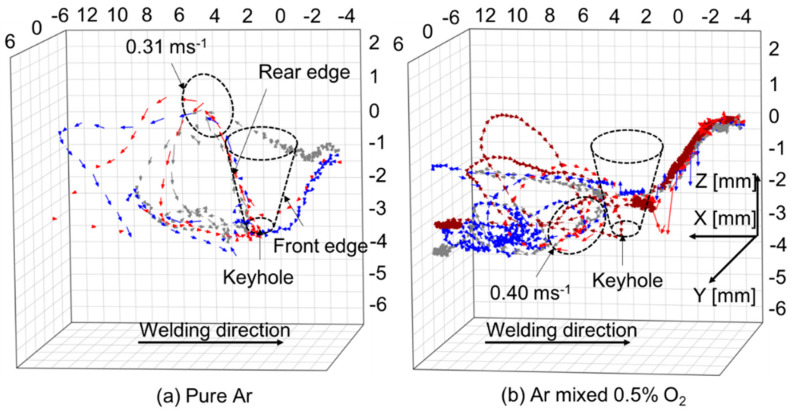
Three-dimensional convective patterns inside the weld pool for different shielding gas compositions [69].

Figure 16 shows 3D convective patterns inside the weld pool for different shielding gas compositions [69]. The weld bead appearances and cross sections of the weld beads have already been presented in Figure 6. It was presented that in the case of pure Ar shielding gas, the molten metal flowed upward behind the keyhole. In the case of Ar with 0.5% O_2_ shielding gas, the flow direction became downward behind the keyhole. The molten metal flow in the weld pool is varied depending on the keyhole shape, for example, by the keyhole diameter and keyhole wall inclination angle. The keyhole diameter is large on the top side and small on the bottom side in the pure Ar case, but it is smaller on the top side and larger on the bottom side in the Ar mixed with 0.5% O_2_ case. The magnitude and direction of the shear force are influenced by this difference. As a result, it was found that the addition of a small amount of oxygen into Ar shielding gas assuming shielding failure strongly affected the heat transport process. It is also implied that narrow and deep penetration can be achieved by mixing oxygen slightly such as in the AA-TIG welding process [82]. This mechanism is considered to be related primarily to the variation in shear force because of different keyhole shape formation due to change in the surface tension rather than the variation in the magnitude and direction of the Marangoni force.

### 4.4. Effect on Microstructure of Solidified Metal in Welded Joint

As mentioned above, the heat and mass transport within the weld pool governs the formation of the temperature field in the weld pool and therefore has a strong influence on the microstructure of solidified metal, mainly according to a temperature history during the cooling process, thus affecting mechanical properties of the welded joint. 

There are many previous studies on the microstructure in KPAW. For example, microstructures when applying KPAW to various materials have been reported, such as duplex stainless steel [83,84], Inconel [85], aluminum alloy [86], and titanium alloy [87]. Comparative studies with microstructures in other arc welding processes have also been conducted. Gupta et al. compared the welded joints prepared by a single-pass KPAW and multi-pass GATAW of M 250 (Maraging steel) with a thickness of 8 mm, showing improved mechanical and metallurgical properties for KPAW in comparison to those for GTAW [88]. Welding parameters such as current waveform also affect the microstructure [85,87]. Kumar et al. found that pulse current helps in grain refinement, which leads to a higher ultimate tensile strength of the welded joints [87]. Large current and high peak current lead to more heat and a longer cooling time, resulting in coarse grain. When the pulse rate is increased, the tensile strength is also increased.

**Figure 17:**
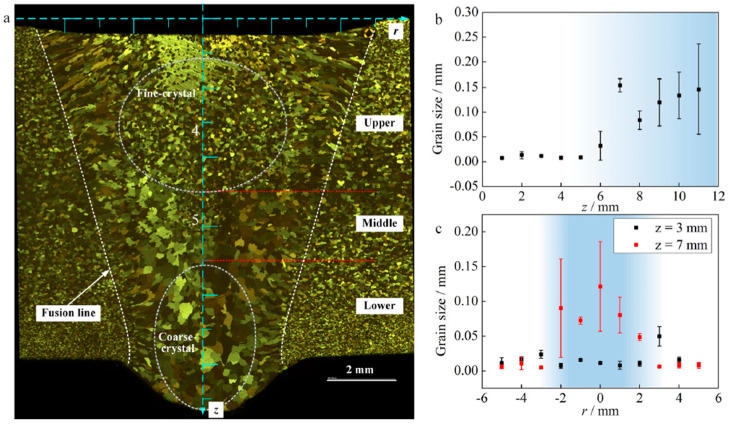
Microstructure and crystal size of the weld bead in VPPAW of a thick aluminum plate [71].

The above studies basically demonstrate the relationship between welding conditions and microstructures experimentally. Although these results are very useful industrially, its mechanism has not been fully elucidated academically. In particular, there are very few studies on the formation process of the microstructure in welded joints based on quantitative discussions of heat and mass transport within the weld pool. Yan et al. investigated the gravity effect on the mechanical property of VPPAW of aluminum alloys at vertical and horizontal positions [89]. Microstructures and residual stress in the welded joint for vertical-up welding were symmetrical. This is in contrast to horizontal welding, which tends to have asymmetric and inhomogeneous microstructures that lead to poor mechanical properties. In the upper side of welded joints for horizontal welding, larger grain sizes brought about lower hardness and lower tensile strength. The difference in the mechanical properties between bilateral sides was suggested to be caused by gravity driving the melting metal flow. After that, Liu et al. carried out the measurements of temperature field and velocity field on the weld pool surface of VPPAW at different positions to discuss the effect of heat and mass transport in the weld pool on microstructure and also mechanical properties of the welded joint through the gravity force [90]. From the measurement, they found that the asymmetric flow of molten metal under the influence of gravity is the main factor leading to uneven temperature distribution and asymmetric grain distribution in the weld pool. Furthermore, Xu et al. clarified the relationship between the microstructure of the welded joint and the heat and mass transport in VPPAW of thick aluminum alloy plates at a flat position by combining in situ three-dimensional X-ray imaging and multi-physics modeling [71]. Figure 17 shows the microstructure and crystal size of the weld bead. It was concluded that the large crystal size observed in the lower layer of the weld is partly caused by heat treatment from the upper layer of the thick plate. An eddy with a high flow velocity to the rear of the weld pool destroys the crystal-growth process, and this is considered to be one of the reasons for fine crystals appearing in the upper part of the weld.

In this way, it has been found that the formation of the microstructure is greatly affected by the flow of the weld pool and the accompanying heat transport. Also, in KPAW, a filler wire is often used when the base material plate thickness is large. Especially when the chemical composition of this filler wire is different from that of the base metal, non-uniform element distribution tends to occur within the weld pool due to weld pool flow [27], which is also thought to affect the formation of the microstructure. Elucidation of the influence of the weld pool formation process on the formation of the microstructure of the solidified metal in the welded joint is still insufficient and remains a future work.

## 5. Summary and Future Work

In this article, the interaction mechanism of the arc, keyhole, and weld pool in KPAW was reviewed. In KPAW, two large eddies are constantly formed in the weld pool behind the keyhole by plasma shear force as the dominant driving force. These govern the heat transport process in the weld pool and have a strong influence on the weld pool formation process. The weld pool flow velocity is much faster than those of other welding processes such as TIG welding and GMA welding, enhancing the heat transport to lower the weld pool surface temperature. Since the strength and direction of this shear force strongly depend on the keyhole shape, it is possible to control the weld pool formation process through changing the keyhole shape by adjusting the torch design and operating parameters. If the lower eddy is relatively stronger, the heat transport to the bottom side increases and the penetration increases. However, burn-through is more likely to occur, and heat transport to the top side decreases, causing undercut. When a small amount of oxygen is added to the shielding gas, the surface tension of the keyhole decreases, making it easier to form a keyhole.

In the above studies, most of them were carried out only for a flat position for simplicity. However, KPAW can also be applied for welding of a pipe or flange that is performed in various welding positions. Accordingly, the direction of gravity acting on the weld pool largely changes during welding. It could critically affect the keyhole formation process, so the interaction mechanism of the arc, keyhole, and weld pool is predicted to change significantly. Furthermore, the effect of the interaction on various welding defects must be investigated more in detail. For example, porosity occurrence is also required to be solved. If gases such as hydrogen are absorbed into the weld pool during welding, those form bubbles. When the bubbles solidify without being released from the weld pool due to flow phenomena including buoyancy, a welding defect called porosity occurs. This is particularly likely to be formed when welding aluminum alloys, causing stress concentration to induce cracks. In order to suppress the porosity, it is necessary to promote the discharge of bubbles out of the weld pool by suitably controlling heat and mass transport processes in weld pool based on the understating of the interaction mechanism. Elucidation of the influence of the weld pool formation process on the formation of the microstructure of solidified metal in the welded joint is still insufficient. The above issues should also be addressed in future works. 

## Data Availability

Data are contained within the article.
